# Protective Effects of Dexmedetomidine on the Vascular Endothelial Barrier Function by Inhibiting Mitochondrial Fission via ER/Mitochondria Contact

**DOI:** 10.3389/fcell.2021.636327

**Published:** 2021-03-11

**Authors:** Han She, Yu Zhu, Haoyue Deng, Lei Kuang, He Fang, Zisen Zhang, Chenyang Duan, Jiaqing Ye, Jie Zhang, Liangming Liu, Yi Hu, Tao Li

**Affiliations:** ^1^Department of Anesthesiology, Daping Hospital, Army Medical University, Chongqing, China; ^2^State Key Laboratory of Trauma, Burns and Combined Injury, Second Department of Research Institute of Surgery, Daping Hospital, Army Medical University, Chongqing, China

**Keywords:** sepsis, Drp1, ER-MITO contact, dexmedetomidine, vascular endothelial barrier function

## Abstract

The damage of vascular endothelial barrier function induced by sepsis is critical in causing multiple organ dysfunctions. Previous studies showed that dexmedetomidine (Dex) played a vital role in protecting organ functions. However, whether Dex participates in protecting vascular leakage of sepsis and the associated underlying mechanism remains unknown yet. We used cecal ligation and puncture induced septic rats and lipopolysaccharide stimulated vascular endothelial cells (VECs) to establish models *in vivo* and *in vitro*, then the protective effects of Dex on the vascular endothelial barrier function of sepsis were observed, meanwhile, related mechanisms on regulating mitochondrial fission were further studied. The results showed that Dex could significantly reduce the permeability of pulmonary veins and mesenteric vessels, increase the expression of intercellular junction proteins, enhance the transendothelial electrical resistance and decrease the transmittance of VECs, accordingly protected organ functions and prolonged survival time in septic rats. Besides, the mitochondria of VECs were excessive division after sepsis, while Dex could significantly inhibit the mitochondrial fission and protect mitochondrial function by restoring mitochondrial morphology of VECs. Furthermore, the results showed that ER-MITO contact sites of VECs were notably increased after sepsis. Nevertheless, Dex reduced ER-MITO contact sites by regulating the polymerization of actin via α_2_ receptors. The results also found that Dex could induce the phosphorylation of the dynamin-related protein 1 through down-regulating extracellular signal-regulated kinase1/2, thus playing a role in the regulation of mitochondrial division. In conclusion, Dex has a protective effect on the vascular endothelial barrier function of septic rats. The mechanism is mainly related to the regulation of Drp1 phosphorylation of VECs, inhibition of mitochondrial division by ER-MITO contacts, and protection of mitochondrial function.

## Introduction

Sepsis is a life-threatening organ dysfunction caused by a dysregulated host response to infection (Jawad et al., 2012; Peake et al., 2014). The damage of vascular endothelial barrier function is a critical pathophysiology process during the development of sepsis (Koh et al., 2010), which will lead to multiple organ dysfunction syndrome (MODS) with high mortality. The vascular endothelium is an important organ of the body, which constitutes the fundamental barrier between blood and tissues (Page and Liles, 2013; Zheng et al., 2020). However, there is still lack of targeted prevention and treatment of vascular leakage after sepsis. Therefore, it is of great clinical significance to look for effective measures to improve sepsis-induced vascular endothelial barrier dysfunction.

Dexmedetomidine (Dex) is a highly selective α_2_-adrenoceptor agonist with sedative, analgesic, and anxiolytic effects (Nelson et al., 2003). Compared with midazolam or propofol, Dex can simulate “natural sleep” without distinct respiratory depression and is suitable for critically ill patients. Previous studies showed that Dex had a crucial protective effect on the damage of many organs, such as lungs and intestines, by inhibiting inflammation and regulating oxidative stress (Sha et al., 2019). However, whether Dex could protect the vascular endothelial barrier function in sepsis and how it works remains obscure.

Dysfunction of mitochondria is a major factor contributing to organ failure, and the dysfunction degree of mitochondria is directly related to the outcome of patients (Brooks et al., 2009; Liu et al., 2012; Roy et al., 2019). Recent studies found that Dex could protect organ function by recovering mitochondrial function. For example, Dex could protect against cerebral ischemia-reperfusion injury by activating mitochondrial ATP potassium channels (Yuan et al., 2017). Additionally, basic research demonstrated that the dynamic balance of mitochondria, including mitochondrial fission and fusion, ensured mitochondrial function maintenance under physiological conditions. While pathological stimulation could induce overactive fission of mitochondria (Hall et al., 2013), which seriously impairs mitochondrial function, resulting in decreased ATP production, cellular calcium disorder, and mPTP opening. However, it is still unknown whether Dex can protect the vascular endothelial barrier function of septic rats by regulating mitochondrial fission.

Herein, we used the cecal ligation and puncture (CLP) induced septic rats and lipopolysaccharide (LPS) stimulated vascular endothelial cells (VECs) to establish models *in vivo* and *in vitro*, and explored the protective effect of Dex on the vascular endothelial barrier function of sepsis and the underlying mechanism associated with mitochondrial fission.

## Materials and Methods

### Ethical Approval of the Study Protocol

All procedures were performed under the guidelines for the Care and Use of Laboratory Animals published by the US National Institutes of Health and were approved by the Laboratory Animal Welfare and Ethics Committee of the Army Medical University (No. DHEC-2012-069). Sprague-Dawley (SD) rats were purchased from the Animal Center of the Research Institute of Surgery.

### Reagents

Dexmedetomidine was purchased from Hengrui (Jiangsu, China). Albumin-fluorescein isothiocyanate conjugate (FITC-BSA), Evans Blue, and Lipopolysaccharide (LPS) were purchased from Sigma (St. Louis, MO, United States). Antibodies for Drp1, ZO-1, VE-cadherin, Occludin, β-actin, ANT, and ROS Detection Kit were purchased from Abcam (Cambridge, MA, United States). Antibodies for phospho-Drp1 (Ser616 and Ser637), ERK1/2, phospho-ERK1/2 were purchased from Cell Signaling Technology (Danvers, MA, United States). Mito-Tracker and ER-Tracker were purchased from Thermo Fisher Scientific (Waltham, MA, United States). Mitochondria Isolation Kit was purchased from Invent Biotechnologies, Inc. (Beijing, CHINA). Atipamezole was purchased from MedChemExpress (Monmouth, NJ, United States). All other chemicals were purchased from Sigma unless specifically mentioned otherwise.

### Animals Preparation and Sepsis Model

Adult male and female Sprague-Dawley (SD) rats (200-220 g) were anesthetized with sodium pentobarbital (45 mg/kg intraperitoneal). CLP induced the sepsis model of rats with aseptic methods as described previously (Zhu et al., 2016). Briefly, alaparotomy was performed, and then the cecum was exposed and ligated. The hole was punctured 0.7 cm from the distal end with a triangular needle (the needle was approximately 1.5 mm indiameter). Feces were allowed to flow into the abdominal cavity. After closure of the abdomen, the rats were returned to the cages and allowed food and water *ad libitum*.

### Cell Preparation

Vascular endothelial cells were obtained from pulmonary veins of SD rats, as described previously (Zhao et al., 2020). Rats were anesthetized and sterilized with iodine, and then rats received thoracotomy. The pulmonary veins were separated from hilus pulmonis after the heart was cut off. After washed with sterile PBS for 3 times, the veins were sheared to pieces, and attached to the bottom of the culture flask with 5 mL ECM (Scicell, America; 5% fetal bovine serum) medium. 3 days later, the pieces were removed from the flask, and the cells crawling on at the bottom of the culture flask were VECs, and the 3–5 passage of VECs were used in the following study.

### FITC-BSA Leakage of Mesenteric Microvessels

Rats were anesthetized, and the ileocecal portion of the mesentery was exposed and placed in a transparent stage. The mesentery was moisturized with 37°C saline throughout the whole procedure to keep it warm and moist. Then rats were injected with FITC-BSA (9 mg/kg) intravenously. 6 min after basal observation, fluorescence intensity of FITC-BSA in mesenteric microvessels was measured at 0, 1, 3, and 6 min by inverted intravital microscopy (HAMAMATSU, Japan).

### Measurement of Pulmonary Vascular Permeability in Rats With FITC-BSA/Evans Blue

Rats were anesthetized, and FITC-BSA (9 mg/kg) or Evans blue (60 mg/kg) was injected through the jugular vein. The abdomen was opened along with the linea alba abdominis after 1 h of FITC-BSA (or 30 min after Evans blue) administration. The abdominal aorta was cut, and the phosphate buffer solution (PBS) was perfused through the jugular vein. The left lung’s upper lobe was dried and weighed, and PBS was added and homogenized in an ice-bath. The homogenate was transferred to a centrifuge (8,000 g, 4°C, 10 min), and the supernatant was then centrifuged again (16,000 g, 4°C, 10 min). The optical density (OD) of the supernatant was determined with a spectrophotometer [excitation wave length: 562 nm (Evans blue)]. Besides, the protein concentration of the supernatant was detected with the BCA protein assay kit (Thermo Fisher Scientific). Finally, the OD value ratio to the protein concentration was considered the pulmonary vascular permeability. Right lung tissue was embedded in Optimal Cutting Temperature compound, and frozen sections (10–20 mm thickness) were generated. The infiltration of FITC-BSA in the lung was observed by a laser confocal microscope (Leica SP5, Germany). The mean optical density was considered to reflect the infiltration of FITC-BSA in the lung. In the Evans blue group, the lung tissue was photographed with a camera (Pentax K7) before homogenization.

### Transendothelial Electrical Resistance(TER) and FITC-BSA Leakage of VECs

VECs were seeded on six-well, 3 μm cell culture inserts (BD Biosciences, Franklin Lakes, NJ, United States), and TER of VECs was assessed by Voltohmmetre (World Precision Inc., America) every 30 min. The TER value measured in the non-cell chamber was regarded as blank control. The resistivity of VECs = (actual TER-blank control)/actual TER. FITC-BSA leakage of VECs was measured after TER analysis, FITC-BSA (10 μg/mL) was added into upper inserts of the transwell, and 200 μL of the medium of the lower chamber at 10, 20, 30, 40, 50, and 60 min was collected for the measurement of fluorescence intensity. An equal volume of culture medium was added into the lower chamber after medium each collection. FITC-BSA leakage (%) = (A10 + A20 + A30 + A40 + A50 + A60)/total fluorescence intensity, and Ax represented the fluorescence intensity at x min.

### Transmission Electronic Microscopy Observation

Fresh pulmonary veins were quickly fixed with arsenate buffer containing 2.5% glutaraldehyde (pH = 7.4, 4°C) for 24 h. After three 5 min-wash with 0.13 M PBS, the veins were postfixed in 1% OsO_4_ for 2 h at room temperature and then dehydrated in a graded ethanol series (65, 70, 75, 80, and 95% for 10 min each) (Duan et al., 2020). After that, the veins were incubated with tert-butoxide for 10 min and then dried with CO_2_, stained with uranyl acetate, coated with gold (Au) using an ion sputter coater. Finally, samples were viewed and imaged with a transmission electron microscope (TEM) (H-7700, Hitachi Company, Japan).

### Respiratory Control Ratio Detection

Fresh pulmonary veins were quickly fixed with arsenate buffer. Freshly heart, liver, kidney, and intestine tissues were put into the separation buffer (sucrose 0.25 mol/L, Na_2_EDTA0.1 mmol/L, Tris0.01 mol/L) for homogenization. The homogenate was centrifuged (4°C, 1,600 g, 12 min), and the supernatant was centrifuged (4°C, 25,000 g, 15 min). The precipitate was collected and resuspended in 1 mL of the separation buffer. The measuring buffer (Tris0.2 mol/L, KCl15 mmol/L, KH_2_PO_4_15 mmol/L, Na_2_EDTA1 mmol/L, MgCl_2_5 mmol/L, sucrose 0.25 mol/L) was heated to 30°C, and then added 0.2 mL of the mitochondrial mixture to the reaction chamber and equilibrated for 20 s, and finally added 10 μL sodium malate and 10 μL sodium glutamate and 5 μL ADP in sequence. The respiratory control ratio was measured by the mitochondrial dissolved oxygen meter (MT 200, Stranthkelvin, United Kingdom).

### Mitochondrial Morphology Observation by Immunofluorescence

VECs were seeded in the confocal chamber and incubated with Mito-tracker (1:10,000) for 30 min at 37°C. Mitochondrial morphology was visualized using confocal laser scanning microscopy (Leica SP5, Germany). The length of mitochondria was analyzed by Image J software^1^, which contains a Mitochondrial Network Analysis (MiNA) toolset (Valente et al., 2017).

### Prediction of ssKSRs With GPS

The prediction of the Drp1 phosphorylation network and site-specific kinase-substrate relations (ssKSRs) were conducted by a software package of iGPS (GPS algorithm with the interaction filter)^2^. Protein kinases are classified into four levels, including group, family, subfamily, and single kinase. In total, GPS contains 144 and 69 individual predictors to predict ssKSRs from primary sequences of proteins for serine/threonine kinases (STKs) and tyrosine kinases (TKs), respectively (Wang et al., 2020).

### Statistical Analysis

Statistical analyses were performed using SPSS 17.0 (SPSS Inc., Chicago, IL, United States). Data are presented as means ± SD of at least three independent experiments. One-way analysis of variance and *post hoc* test (S-N-K/LSD) were used to analyze the difference between experimental groups. Survival time was analyzed by the median and interquartile range and Kaplan-Meier survival analyses and the log-rank test. Values of *p <* 0.05 were considered statistically significant.

## Results

### Protective Effects of Dexmedetomidine on the Vascular Permeability in Septic Rats

**FIGURE 1:**
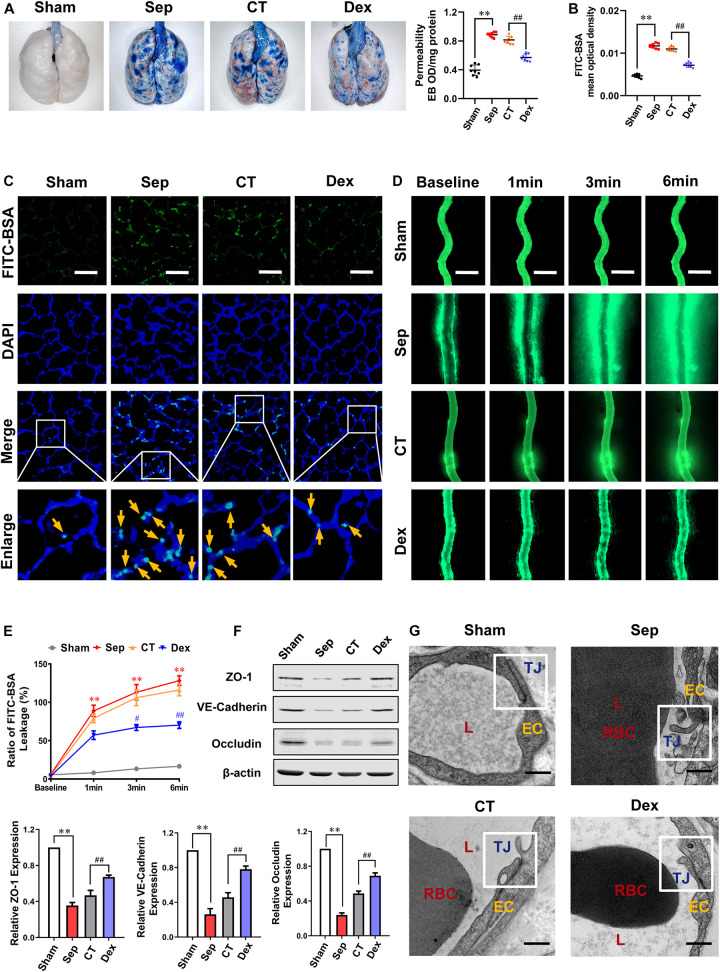
Protective effect of dexmedetomidine on vascular permeability of septic rats. **(A)** Vascular permeability of the lung, measured by the leakage of Evans Blue, *n* = 8. **(B,C)** Vascular permeability of the lung, measured by the mean optical density of intravenously injected FITC-BSA *in vivo* (Bar, 50 μm), *n* = 8. **(D,E)** The FITC-BSA leakage of mesentery microvessels in rats, dynamically measured by intravital microscopy *in vivo* (Bar, 100 μm), *n* = 8. **(F)** Western blot analysis of ZO-1, Occludin and VE-cadherin in the superior mesenteric vein of rats treated with CLP, *n* = 3. **(G)** Representative transmission electron microscope microphotographs of tight junctions of pulmonary venules (Bar, 200 nm), *n* = 8. EC, endothelial cell; RBC, red blood cell; TJ, tight junction; L, lumen; Sham, sham group; Sep, sepsis group; CT, conventional treatment group; Dex, dexmedetomidine group. ^∗∗^*P* < 0.01, as compared with sham group; ^##^*P* < 0.01, as compared with CT group; ^#^*P* < 0.05, as compared with CT group.

In order to observe the effect of Dex on vascular endothelial barrier function, the permeability of lung and intestinal vascular to FITC-BSA or Evans blue following sepsis and the expression of intercellular junction proteins were measured. According to the guidelines for sepsis, after 12 h of CLP, rats received fluid resuscitation for 3 h with lactated Ringer’s solution (LR) (35 mL/kg), vasopressor (dopamine5∼10 μg/kg/min), and antibiotic (cefuroxime sodium, 100 mg/kg) as the conventional treatment (CT) (Allen et al., 2019). While rats in the Dex group received 10 μg/kg dexmedetomidine 30 min before and 12 h after CLP in addition to receiving CT, and the sepsis group had no treatment. The results showed that the pulmonary vascular permeability of septic rats was significantly increased. It appeared the vascular leakage of FITC-BSA (yellow arrows) and Evans blue were increased by 124 and 148% as compared with the sham group (*P* < 0.01). CT did not effectively decrease the pulmonary vascular permeability (*P* > 0.05), while administration of Dex could significantly alleviate the leakage. Compared with the CT group, Evans Blue exudation in the Dex group was decreased by 30% (*P* < 0.01) (Figure 1A), and the leakage of FITC-BSA was decreased by 34.5% (*P*<0.01) (Figures 1B,C).

To further explore the effect of Dex on vascular leakage after sepsis, the mesenteric venule branch was used as a microcirculation representative, the leakage of FITC-BSA from the mesenteric micro-vessels was directly measured through intravital microscopy. Following injecting FITC-BSA (0, 1, 3, 6 min) intravenously, the change of FITC-BSA transmittances was observed. The results showed that the endothelial barrier function of mesenteric microvessels in septic rats was significantly impaired, and the exudation of FITC-BSA was notably increased. A large amount of FITC-BSA leaked out from the blood vessel after 6 min in septic rats (*P* < 0.01). CT did not improve the permeability of mesenteric microvessels effectively (*P* > 0.05) while Dex distinctly alleviated vascular leakage and the FITC-BSA permeability decreased by 39.5% (*P* < 0.01) compared to the CT group (Figures 1D,E).

In general, cell connections such as tight junctions and adhesion junctions play an important role in the permeability of vascular endothelial cells. To investigate the effect of Dex on cell connections in pulmonary vascular endothelial, we observed the changes of tight junctions by the TEM. The results showed that the tight junctions between endothelial cells were closed and dense in the sham group. In the sepsis group, the vascular endothelial cells were swollen, and the tight junctions were obviously damaged, showing a state of relaxation and shedding. CT did not significantly improve the damaged tight junctions, while were restored to a close degree in the Dex group (Figure 1G). There are several key proteins participating in the regulation of vascular leakage, such as tight junction proteins (ZO-1, Occludin), and adherent junction protein (VE-cadherin). The changes of ZO-1, Occludin, and VE-cadherin were further observed. The results showed that the expressions of ZO-1, Occludin, and VE-cadherin in septic rats were distinctly reduced (*P* < 0.01), and CT could not effectively change the decrease of intercellular junction proteins. Following Dex administration, the expressions of ZO-1, Occludin, and VE-cadherin were evidently improved, increasing by 60.3, 41.9, and 70.6%, respectively (*P* < 0.01) (Figure 1F). These results indicate that Dex has a protective effect on the vascular endothelial barrier function of septic rats.

### Protective Effects of Dexmedetomidine on the Permeability of VECs After Sepsis

**FIGURE 2:**
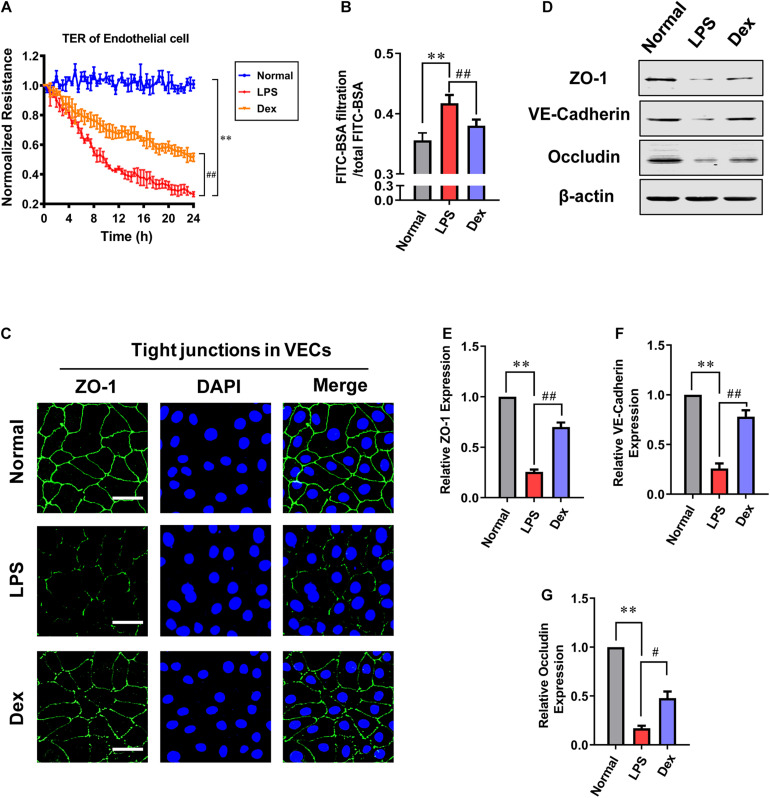
The influence of dexmedetomidine on the permeability of vascular endothelial cells after sepsis. **(A)** Effects of dexmedetomidine on the TER (transendothelial electrical resistance) of VEC monolayers after sepsis, *n* = 3. **(B)** Effects of dexmedetomidine on the infiltration rate of FITC-BSA in monolayer VECs after sepsis, *n* = 3. **(C)** Measurement of the expression of ZO-1(green) after sepsis in VECs by immunofluorescence (Bar, 25 μm), *n* = 3. **(D–G)** Western blot analysis of ZO-1, Occludin and VE-cadherin in VECs after sepsis, *n* = 3. Normal: nomal group; LPS: lps group; Dex: dexmedetomidine group. ^∗∗^*P <* 0.01, as compared with normal group; ^##^*P <* 0.01, as compared with lps group; ^#^*P* < 0.05, as compared with lps group.

To further investigating the effect of Dex *in vitro*, the permeability of VECs following LPS stimulation was measured. Cells were cultured till the confluence reached 60–70%, and then LPS (1 μg/mL) was incubated for 12 h in the sepsis group. The Dex group was incubated with 0.1 μM dexmedetomidine for 30 min before adding LPS (1 μg/mL). The results showed that the transmembrane electrical resistance (TER) of VEC decreased by 73.7% after LPS stimulation compared with the normal group. Meanwhile, the BSA leakage of VECs obviously increased (*P* < 0.01). Dex could notably improve the permeability of VECs. Compared with the LPS group, TER increased by 94.1%, and the BSA leakage decreased by 8.9% in VECs of the Dex group (*P* < 0.01) (Figures 2A,B). Next, we observed the intercellular junction proteins expressions of VECs. The immunofluorescence results revealed that the ZO-1 was distributed continuously along the vascular endothelial cell membrane in the normal group, while in the LPS group, the fluorescence intensity of ZO-1 was weakly expressed and loosely distributed with gaps (Figure 2C), and the expressions of ZO-1, VE-cadherin, and Occludin, respectively, decreased by 74.3, 74, and 83.06% as compared to the normal group (*P* < 0.01) (Figures 2D–G). Following Dex administration, the distribution of ZO-1 was improved with complete structure and clear boundary; simultaneously, in comparison with the LPS group, the expression of ZO-1, VE-cadherin, and Occludin were increased by 172.8, 200% (*P* < 0.01), and 182.3% (*P* < 0.05), respectively (Figures 2D–G). These results further suggest that Dex has a protective effect on the vascular endothelial barrier function of septic rats.

### Effects of Dexmedetomidine on Mitochondrial Fission and Mitochondrial Functions After Sepsis

**FIGURE 3:**
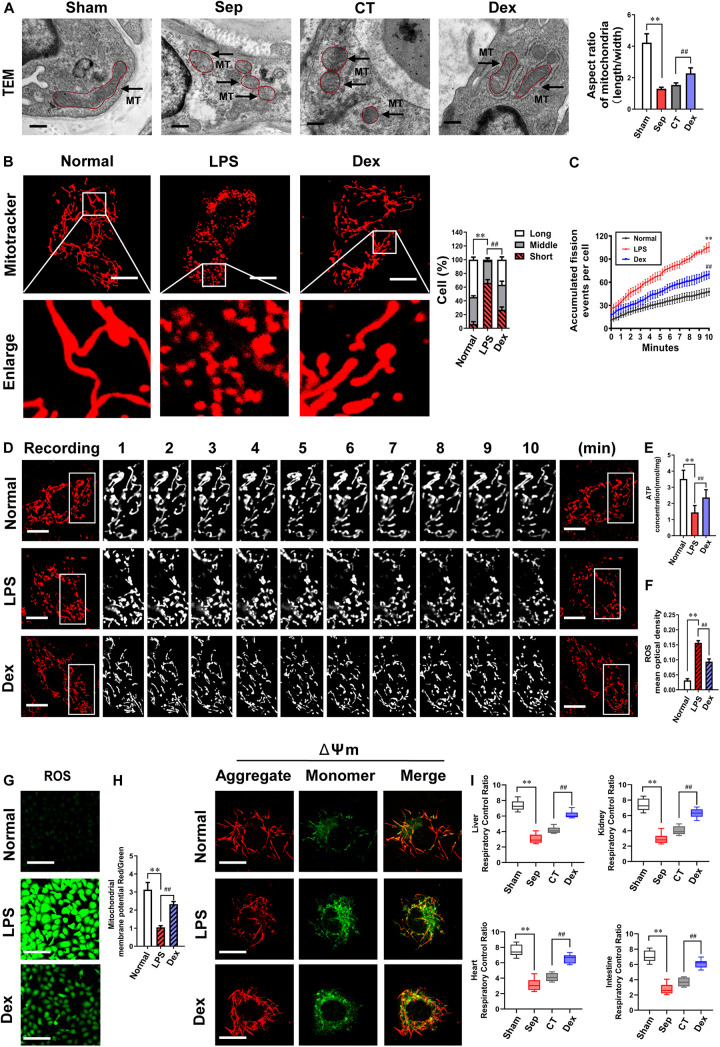
The effects of dexmedetomidine on mitochondrial fission and mitochondrial functions after sepsis. **(A)** TEM (transmission electronic microscopy) images to observe mitochondrial morphology of pulmonary venules in septic rats (Bar, 200 nm), *n* = 8. **(B)** Confocal images to observe mitochondrial morphology of VECs after sepsis (Bar, 25 μm), *n* = 50. **(C,D)** Time-lapse images of mitochondrial morphologic alternation of VECs per 15 s after sepsis by confocal immunostaining (Bar, 20 μm), *n* = 3. **(E)** Effects of dexmedetomidine on the ATP of VECs after sepsis, *n* = 3. **(F–H)** Representative confocal images of ROS(Bar, 100 μm) and △Ψm (Bar, 25 μm) after sepsis in VECs, *n* = 3. **(I)** Effects of dexmedetomidine on the respiratory control ratio in septic rats, *n* = 3. Sham: sham group; Sep: sepsis group; CT: conventional treatment group; Normal: nomal group; LPS: lps group; Dex: dexmedetomidine group. ^∗∗^*P <* 0.01, as compared with normal group; ^##^*P <* 0.01, as compared with lps group.

Mitochondria are dynamic organelles that undergo remodeling via fusion and fission. The fine balance between these two opposing processes determines mitochondrial morphometric properties and functions. Excessive mitochondrial fission leads to mitochondrial and organ dysfunction. Whether Dex exerts a protective effect on vascular endothelial barrier function by regulating mitochondrial fission is unclear. In the present study, we examined the morphologic changes of mitochondria in pulmonary veins with a TEM and found that the number of mitochondria substantially increased, and the aspect ratio of mitochondria decreased by 69.3% after sepsis (*P* < 0.01). The mitochondrial morphology did not improve, remaining short and small (*P* > 0.05) after CT. In contrast, the mitochondrial morphology was significantly restored, and the aspect ratio increased by 47.7% in the Dex group (*P* < 0.01) (Figure 3A). At the cellular level, the morphological changes of mitochondria were further observed by a laser confocal microscope. Each group randomly selected 50 cells and the mitochondrial morphology was blindly scored and classified into three categories: Long (>6 μm), Middle (3–6 μm), Short (<3 μm) (Gao et al., 2017). The results showed that the mitochondria were mostly in elongated shapes under normal conditions, of which 54% were long mitochondria, 40% were medium mitochondria, and only 6% were short. However, the proportion of short mitochondria distinctly increased and reached 66% after LPS stimulation. Dex treatment could recover the long mitochondria accounted for 37% and the proportion of short reduced to 26% (Figure 3B), indicating that Dex could effectively alleviate mitochondrial fission. Since mitochondrial morphology is subject to changes in the frequency of division and fusion, we further continuously observed 10 min to record the frequency of mitochondrial divisions by confocal immunostaining, and it was found that the frequency of mitochondrial divisions increased by 121% after sepsis (*P* < 0.01), while compared with the LPS group, the frequency of mitochondrial divisions after Dex treatment decreased by 33.8% (*P* < 0.01) (Figures 3C,D).

Next, we explored whether Dex can protect mitochondrial function by protecting mitochondrial morphology. Mitochondrial membrane potential (ΔΨm) is a vital part that directly affects the mitochondrial capacity, and the generation of ROS is closely related to the functional status of mitochondria. Therefore, we observed ΔΨm, ROS, and ATP production of VECs. The results showed that compared with the normal group, ΔΨm and ATP levels were significantly reduced, and the generation of ROS was increased after 12 h LPS treatment (*P* < 0.01). Meanwhile, ΔΨm and ATP levels in the Dex group, respectively, increased by 121.7 and 64.7%, and ROS decreased by 40% (*P* < 0.01) in comparison with the LPS group (Figures 3E–H). In addition to VECs, Dex also protected the mitochondrial functions of heart, liver, kidney, and intestinal mucosal epithelial cells *in vivo*. The respiratory control rates (RCRs) of liver, kidney, heart, and intestine tissues were distinctly reduced, and all of the reduction rates were higher than 55% (*P* < 0.01). RCRs were not improved significantly after CT (*P* > 0.05), while Dex could effectively restore the above parameters. Compared with the CT group, RCRs of liver, kidney, heart, and intestine tissues increased by 47.4, 55.8, 58.7, and 66.7%, respectively (*P* < 0.01) (Figure 3I). These results suggest that Dex can protect the vascular endothelial barrier function by alleviating excessive mitochondrial division and protecting mitochondrial function.

### Dexmedetomidine Reduced ER-MITO Contacts via Actin Polymerization Inhibition and Thus Inhibited Mitochondrial Fission

**FIGURE 4:**
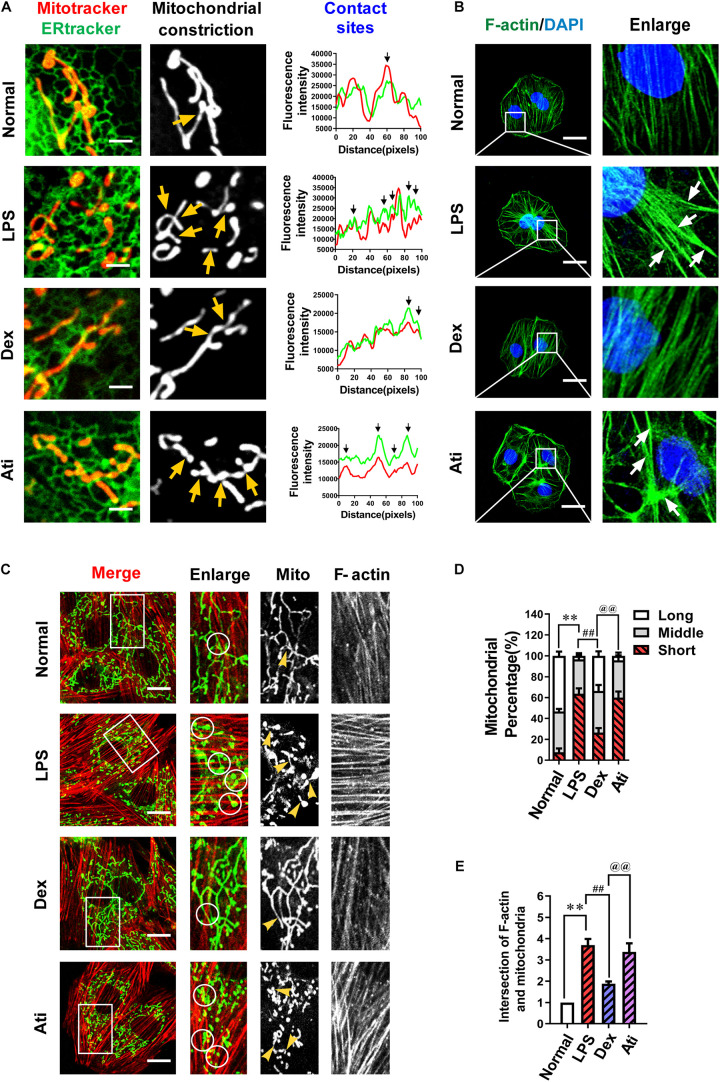
The effects of dexmedetomidine on F-actin and ER-MITO contact of vascular endothelial cells after sepsis. **(A)** Representative fluorescent images of ER-MITO contact transformed with ERtracker and Mitotracker in VECs after sepsis (Bar, 5 μm), *n* = 3. The sites of mitochondrial fission (yellow arrows) correspond to black arrows of contact sites on the line scan. **(B)** Confocal images to observe the effect of dexmedetomidine on the polymerization of actin (Bar, 25 μm), *n* = 3. **(C)** Confocal images to observe mitochondria and F-actin of VECs after sepsis(Bar, 20 μm), *n* = 3. **(D)** The statistical analysis of mitochondrial morphology of VECs in different groups. Quantitation was performed in triplicate and scored into three categories: long, middle, and short mitochondria, with 50 cells scored per group. **(E)** The intersections of F-actin and mitochondria of VECs in different groups were calculated by Image J software. N, nomal group; LPS, lps group; Dex, dexmedetomidine group; Ati, Atipamezole group. ^∗∗^*P <* 0.01, as compared with normal group; ^##^*P <* 0.01, as compared with lps group; ^@@^*P <* 0.01, as compared with dex group.

Previous studies demonstrated (Wang et al., 2002; Wu et al., 2016; Chakrabarti et al., 2018) that endoplasmic reticulum and mitochondria (ER-MITO) contact played a vital role in mitochondrial fission by inducing mitochondrial pre-contraction before mitochondrial fission. However, whether Dex’s regulation of mitochondrial division is related to ER-MITO contact has never been shown. Firstly, we used immunofluorescence to observe the effect of Dex on ER-MITO contact. The results found that LPS stimulation induced mitochondria to split, and the ER-MITO contact sites were significantly increased with evident mitochondrial pre-constriction traces compared with the normal group. After the treatment with Dex, the number of ER-MITO contact sites and mitochondrial pre-constriction were both reduced distinctly compared with the LPS group, showing a similar trend as the inhibition of mitochondrial division (Figure 4A).

The process of actin accumulated to bundles (F-actin) and pull the endoplasmic reticulum and result in ER-MITO contact was a vital initiation step in the mitochondrial pre-constriction. To explore whether the increase in ER-MITO contact caused by Dex is related to F-actin, we further observed the effect of Dex on actin polymerization. The results showed that compared with the normal group, F-actin stress fibers increased notably after the stimulation of LPS and aggregated to form bundles (white arrows) in VECs, while Dex could inhibit actin aggregation from forming stress fibers (Figure 4B). The relationship between actin aggregation and mitochondrial division was further observed with a laser confocal microscope. The contacts between F-actin and mitochondria increased about 3–4 times after LPS stimulation as compared with the normal group (*P* < 0.01) (white circles). Meanwhile, short mitochondria increased from 7% in the normal group to 64% in the LPS group. In the Dex group, the contacts between F-actin and mitochondria were evidently reduced, and short mitochondria decreased to 26% (*P* < 0.01) (Figures 4C–E).

Since Dex is a highly selective α_2_ adrenergic receptor agonist, we used the targeted blocker atipamezole (Ati) to illustrate whether Dex exerted the above effects through regulating α_2_ receptors. 1 μM atipamezole was added to incubate the cells 10 min before adding 0.1 μM dexmedetomidine, and the results were consistent with expectations. Ati could significantly antagonize the polymerization of actin caused by Dex and the increase of ER-MITO contact sites, thereby antagonizing the protective effect of Dex on excessive mitochondrial division (Figures 4A–E). These results suggested that Dex exerted the protective role in mitochondrial function by inhibiting actin polymerization via α_2_ receptors activation, reducing the number of ER-MITO contact sites, and suppressing mitochondrial division.

### Dexmedetomidine Phosphorylates ERK1/2-Mediated Mitochondrial Fission

**FIGURE 5:**
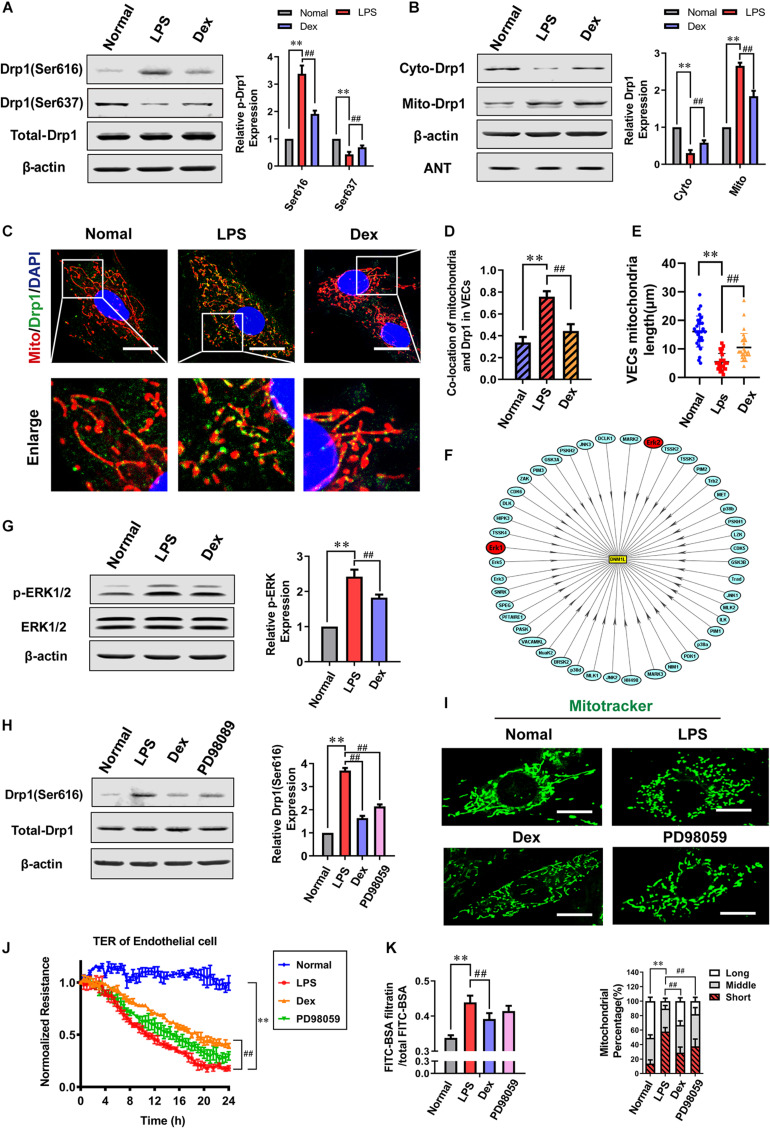
Dexmedetomidine regulates ERK1/2-mediated mitochondrial fission. **(A)** Effects of dexmedetomidine on the phosphorylation of Drp1 after sepsis in VECs detected by Western Blotting, *n* = 3. **(B)** Effects of dexmedetomidine on the mitochondrial translocation of Drp1 after sepsis in VECs, *n* = 3. **(C,D)** The co-location of Drp1 and mitochondria of VECs in each group (Bar, 25 μm), *n* = 3. **(E)** The length of mitochondria of VECs in each group. **(F)** Drp1 protein phosphorylation network (PPN) predicted by iGPS 1.0 software. **(G)** Effects of dexmedetomidine on the phosphorylation of ERK1/2 after sepsis in VECs, *n* = 3. **(H)** Effects of dexmedetomidine and ERK1/2 inhibitor on the phosphorylation of ERK1/2 after sepsis in VECs, *n* = 3. **(I)** Mitochondrial morphology in PD98059-treated VECs after sepsis (Bar, 15 μm), *n* = 3. **(J,K)** Effects of PD98059 on the TER and FITC-BSA infiltration rate of VEC monolayers after sepsis, *n* = 3. Normal, normal group; LPS, lps group; Dex, dexmedetomidine group. PD98059: ERK1/2 inhibitor group. ^∗∗^*P*< 0.01, as compared with normal group; ^##^*P* < 0.01, as compared with lps group.

Based on the pre-constriction of mitochondria caused by ER-MITO contact, Dynamin-related protein 1 (Drp1) is recruited to mitochondria and then cut mitochondria through GTPase, which is a vital link that leads to mitochondrial fission. As a member of the dynamin superfamily, Drp1 has many phosphorylation sites with various roles. The Ser 616 and Ser637 sites are critical for the translocation of Drp1 from the cytoplasm to mitochondria. Western Blotting results showed that there was no obvious difference in total expression of Drp1 after LPS stimulation compared to the normal group while the phosphorylation of Drp1 Ser616 increased by 238.3% (*P* < 0.01), and the phosphorylation of Ser637 decreased by 57% (*P* < 0.01). Moreover, the expression of Drp1 increased in mitochondria and decreased in cytoplasm significantly after sepsis (*P* < 0.01) (Figures 5A,B), and the Adenine nucleotide translocase (ANT) was used as a reference when detecting the change of Mito-Drp1. The confocal images indicated that the co-localization ratio between Drp1 and mitochondria distinctly increased, and the length of mitochondria shortened from 16.10 ± 5.43 μm in the normal group to 5.47 ± 2.91 μm after sepsis (*P* < 0.01) (Figures 5C–E). Compared with the LPS group, the phosphorylation level of Ser616 reduced by 43.5%, and the level of Ser637 increased by 61.1%. The mitochondrial translocation of Drp1 reduced, and the expression of Drp1 in cytoplasm notably increased in the Dex group (*P* < 0.01) (Figures 5A,B). Furthermore, the co-localization ratio reduced, and the mitochondrial length was 10.86 ± 5.91 μm in the Dex group (*P* < 0.01) (Figures 5C–E). We further observed the effect of Dex on mitochondrial fusion proteins and found no significant differences in the expressions of OPA1, Mfn1, and Mfn2 following Dex administration (Supplementary Figure 1). Overall, these results suggested that Dex could effectively inhibit the activation and mitochondrial translocation of Drp1, and influence the morphology and function of mitochondria.

To further explore the mechanism of Dex regulating drp1 activation, a software package *in vivo* GPS (iGPS 1.0) (Wang et al., 2020) was used to predict the site-specific kinase for all phosphorylation-sites of Drp1. The prediction results showed that the extracellular regulated protein kinases1/2 (ERK1/2) could significantly regulate the Ser616 site of Drp1 (Figure 5F and Table 1), and previous studies (Zhao et al., 2019; Zhang et al., 2019) also reported that ERK1/2 is an essential kinase that regulates Drp1. So the effect of ERK1/2 on Dex regulating Drp1 Ser 616 phosphorylation was further studied. The results pointed out that the phosphorylation of ERK1/2 increased by 142.4% in the LPS group (*P* < 0.01), and Dex could prominently reduce the ERK1/2 phosphorylation (*P* < 0.01) (Figure 5G). Besides, the ERK1/2 inhibitor (PD98059) (10 μM PD98059 was added to incubate the cells 10 min before adding 1 μg/mL LPS) could effectively inhibit the phosphorylation of Drp1 Ser616 (Figure 5H). Furthermore, PD98059 also could inhibit mitochondrial fission (*P* < 0.01) and slightly inhibit vascular leakage (*P* > 0.05) (Figures 5I–K). These results indicate that Dex could reduce mitochondrial division via regulating the activation and translocation of Drp1 by inhibiting the phosphorylation of ERK1/2.

**TABLE 1:** The prediction outcome of Drp1 ser616 and ser637 phosphorylation sites and their site-specific kinase.

ID	Position	Code	Peptide	Gene name	Kinase name	Interaction	Predictor	Score
sp| O00429	616	S	PIPIMPASPQKGHAV	DNM1L	CDK5	String	CMGC/CDK	2.61
sp| O00429	616	S	PIPIMPASPQKGHAV	DNM1L	CDK6	String	CMGC/CDK	2.61
sp| O00429	616	S	PIPIMPASPQKGHAV	DNM1L	PFTAIRE1	String	CMGC/CDK	2.61
sp| O00429	616	S	PIPIMPASPQKGHAV	DNM1L	HIPK3	String	CMGC/DYRK	2.33
sp| O00429	616	S	PIPIMPASPQKGHAV	DNM1L	Erk1	String	CMGC/MAPK	3.47
sp| O00429	616	S	PIPIMPASPQKGHAV	DNM1L	Erk5	String	CMGC/MAPK	3.47
sp| O00429	616	S	PIPIMPASPQKGHAV	DNM1L	Erk3	String	CMGC/MAPK	3.47
sp| O00429	616	S	PIPIMPASPQKGHAV	DNM1L	JNK3	String	CMGC/MAPK	3.47
sp| O00429	616	S	PIPIMPASPQKGHAV	DNM1L	JNK1	String	CMGC/MAPK	3.47
sp| O00429	616	S	PIPIMPASPQKGHAV	DNM1L	p38b	String	CMGC/MAPK	3.47
sp| O00429	616	S	PIPIMPASPQKGHAV	DNM1L	JNK2	String	CMGC/MAPK	3.47
sp| O00429	616	S	PIPIMPASPQKGHAV	DNM1L	Erk2	String	CMGC/MAPK	3.47
sp| O00429	616	S	PIPIMPASPQKGHAV	DNM1L	p38a	String	CMGC/MAPK	3.47
sp| O00429	616	S	PIPIMPASPQKGHAV	DNM1L	p38d	String	CMGC/MAPK	3.47
sp| O00429	637	S	VPVARKLSAREQRDC	DNM1L	SPEG	String	CAMK	2.86
sp| O00429	637	S	VPVARKLSAREQRDC	DNM1L	TSSK4	String	CAMK	2.86
sp| O00429	637	S	VPVARKLSAREQRDC	DNM1L	DCLK1	String	CAMK	2.86
sp| O00429	637	S	VPVARKLSAREQRDC	DNM1L	PSKH2	String	CAMK	2.86
sp| O00429	637	S	VPVARKLSAREQRDC	DNM1L	Trb2	String	CAMK	2.86
sp| O00429	637	S	VPVARKLSAREQRDC	DNM1L	PIM3	String	CAMK	2.86
sp| O00429	637	S	VPVARKLSAREQRDC	DNM1L	Trad	String	CAMK	2.86
sp| O00429	637	S	VPVARKLSAREQRDC	DNM1L	PSKH1	String	CAMK	2.86
sp| O00429	637	S	VPVARKLSAREQRDC	DNM1L	TSSK3	String	CAMK	2.86
sp| O00429	637	S	VPVARKLSAREQRDC	DNM1L	TSSK2	String	CAMK	2.86
sp| O00429	637	S	VPVARKLSAREQRDC	DNM1L	VACAMKL	String	CAMK	2.86
sp| O00429	637	S	VPVARKLSAREQRDC	DNM1L	PIM2	String	CAMK	2.86
sp| O00429	637	S	VPVARKLSAREQRDC	DNM1L	PIM1	String	CAMK	2.86

### Effects of Dexmedetomidine on Survival Time, Survival Rate, and Organ Functions in Septic Rats

**FIGURE 6:**
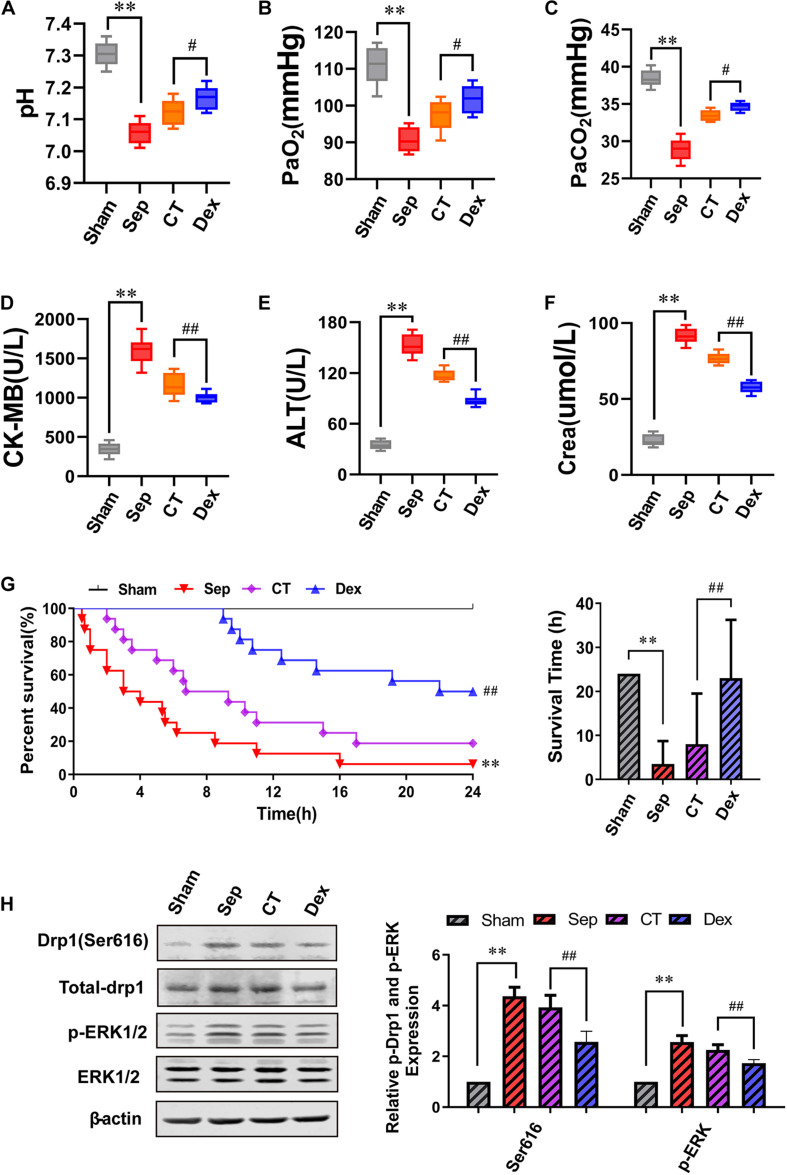
The influence of dexmedetomidine on the survival rate, survival time and organ function of septic rats. **(A–F)** Statistical histogram of pH, PaO_2_, PaCO_2_, ALT, CK-MB, and Crea levels in each group, *n* = 8. **(G)** Survival rate and survival time, *n* = 16. **(H)** Effects of dexmedetomidine on the phosphorylation 616 site of Drp1 and ERK1/2 in septic rats detected by Western Blotting, *n* = 3. Sham, sham group; Sep, sepsis group; CT, conventional treatment group; Dex. dexmedetomidine group. ^∗∗^*P*< 0.01, as compared with sham group; ^##^*P* < 0.01, as compared with CT group; ^#^*P* < 0.05, as compared with CT group.

To evaluate whether Dex can protect organ functions and prolong the survival time of septic rats by alleviating the vascular leakage, we detected the arterial blood gas and the level of CK-MB, ALT, and Crea to reflect the degree of organ damage. We discovered that Dex could remarkably improve the functions of lung, heart, liver, and kidney in septic rats. Compared with the CT group, the pH value was significantly enhanced, and acidosis was alleviated after Dex treatment. Besides, PaO_2_ and PaCO_2_ increased by 4.49 and 3.74%, respectively (*P* < 0.05), while CK-MB, ALT, Crea were decreased by 13.1, 25.4, and 24.7% individually (*P* < 0.01) (Figures 6A–F).

Results showed that the 24 h survival rate of septic rats was only 6.25% (*P* < 0.01), and CT slightly increased the survival rate and survival time compared with the sepsis group. While Dex could significantly improve the survival rate and survival time of septic rats compared with the CT group. The 24 h survival rate was 50%, and the mean survival time was 23 ± 13.25 h in the Dex group (*P* < 0.01) (Figure 6G). In addition, we also verified the mechanism of Dex regulating ERK1/2 mediated Drp1 activation *in vivo*, and the results were consistent with those *in vitro* (Figure 6H). The results suggested that Dex could protect organ functions and prolong survival by inhibiting vascular leakage.

## Discussion

The vascular endothelial barrier dysfunction is the primary cause of sepsis-induced organ dysfunction (Armstrong et al., 2013; Vincent et al., 2013; Miranda et al., 2015; Schmidt et al., 2015). Previous studies found that dexmedetomidine protected against vital organ injury in sepsis by inhibiting inflammatory factors and reducing oxidative stress (Sun et al., 2019; Zi et al., 2019). The present study demonstrated that Dex could effectively alleviate sepsis-induced vascular leakage and vascular endothelial barrier dysfunction in septic rats, thus protect organ functions and ultimately prolong the survival rate and survival time of septic rats, which provided a novel sight for the treatment of sepsis with anesthetic drugs.

Recent studies pointed out that the protective effect of Dex on organ functions may closely related to the regulation of mitochondrial function. At present, it is believed that the main mechanisms to improve mitochondrial function are to activate the mitochondrial ATP-sensitive potassium channel and increase mitochondrial membrane potential (Yuan et al., 2017). This study showed that Dex could suppress the permeability of VECs by protecting mitochondrial morphology. After sepsis, the mitochondrial morphology of VECs is damaged, and mitochondria divided excessively, accompanying ATP production obstacles, ROS production increases, mitochondrial membrane potential, and RCR decreases. However, Dex distinctly inhibited the mitochondrial division of VECs, improved mitochondrial morphology, and protected mitochondrial function. Generally, mitochondria are mostly in elongated or thread shapes in normal condition, while the pathological stimulation lead to fragmentation, furthermore, if the stimuli continue to exist, mitochondria will divide excessively, resulting in reduced ATP production, disturbance of calcium metabolism, and finally develop into mitochondria dysfunction. Numerous studies have confirmed that mitochondrial dysfunction plays a vital role in MODS, and the dysfunction degree is directly related to the outcome of patients (Brooks et al., 2009; Eismann et al., 2009; Liu et al., 2012; Duan et al., 2020). Our study demonstrated that Dex could protect mitochondrial function by improving mitochondrial morphology.

The key links of mitochondrial fission included ER-MITO contact-induced mitochondrial pre-constriction and Drp1-related mitochondria split. Previous studies demonstrated that ER-MITO contact was a functional contact structure formed by the formation of a complex between the endoplasmic reticulum and mitochondria such as IP3R-GRP75-VDAC complex and so on (Friedman et al., 2011). Early reports revealed that the primary function of ER-MITO contact was to regulate the transfer of calcium and lipids between the endoplasmic reticulum and mitochondria. In recent years, researchers have found that ER-MITO contact could participate in various diseases by regulating mitochondrial structure (Rowland and Vorletz, 2012). The studies showed that actin polymerization could increase the formation of ER-MITO contacts (Wales et al., 2016) and promote mitochondrial fission under the effect of Myosin Light Chain Kinase (MLCK) or ROCK. In the present study, we demonstrated that actin in VECs polymerized to form stress fibers after stimulation with LPS for 12 h, which increased ER-MITO contact sites and mitochondrial fission. While Dex could inhibit the aggregation of actin and reduce ER-MITO contact sites, leading to the inhibition of mitochondrial fission. Meanwhile, we also found that atipamezole, the highly selective α_2_ receptor blocker, could significantly antagonize the effects of Dex, suggesting that Dex may inhibit actin polymerization of VECs after sepsis by activating α_2_ receptors. Furthermore, studies (Hatch et al., 2014) have found that actin polymerization can activate the ER-localized inverted formin 2 (INF2), and the activated INF2 aggregated between the mitochondria and the ER, which further increased the formation of ER-MITO contacts. However, whether Dex exerts the same modulation effect on INF2 needs further investigation. Previous studies have shown that increased intracellular Ca^2+^ influx can activate MLCK by mediating Ca^2+^/CaM-PKC and Ca^2+^-cGMP-PKG pathways, up-regulating MLC phosphorylation, causing actin polymerization (Dudek and Garcia, 2001; Duan et al., 2017). A study also found that Dex could reduce calcium influx by activating α_2_ receptors (Zhao et al., 2013). Therefore, we speculated that Dex might inhibit Ca^2+^ influx by activating α_2_ receptors and blocking Ca^2+^/CaM-PKC and Ca^2+^-cGMP -PKG pathway, thereby inhibiting actin polymerization, but the specific mechanism needs to be further studied.

Besides ER-MITO contact, Drp1 activates and translocates from the cytoplasm to the mitochondrial pre-constriction site and cleaves the mitochondria. Present study found that Dex inhibits mitochondrial division through Drp1 phosphorylation. Dex could effectively inhibit the phosphorylation of Drp1 Ser616 in VECs and reduce the translocation of Drp1 from the cytoplasm to the mitochondria. As an important mitochondrial-related protein, Drp1 is of great significance to the regulation of mitochondrial structure and function. Also, Drp1 is known as DNM1L and DLP1, which was first reported in 1997 (Shinh et al., 1997), mainly distributed in the cytoplasm in the form of polymers under physiological condition. The activated Drp1 translocates from the cytoplasm to the mitochondrial outer membrane and polymerizes to form a ring-like structure that enables mitochondrial division. Besides, our prediction of iGPS and previous studies (Jiang H. K. et al., 2014; Prieto et al., 2016) showed that the activity of Drp1 Ser616 is mainly regulated by the ERK1/2 pathway. In the present study, Dex had a significant inhibitory effect on the phosphorylation of ERK1/2 in VECs after sepsis, which is consistent with previous studies (Jiang L. et al., 2014; Qiu et al., 2020). Meanwhile, we confirmed that both Dex and ERK1/2 inhibitor (PD98059) could effectively suppress the phosphorylation of Drp1 Ser616 in VECs, and inhibit vascular leakage, indicating that Dex may regulate the activation and translocation of Drp1 by inhibiting the ERK1/2 phosphorylation pathway. As multiple phosphorylation sites of Drp1 are activated to regulate its function after sepsis, we also observed that Dex could elevate the phosphorylation level of Ser637, which can inhibit the translocation of Drp1. Whether Dex can regulate Drp1 phosphorylation of other sites and the associated mechanism needs to be further studied. The present study demonstrated that Dex alleviated mitochondrial fission for sepsis by two key steps of mitochondrial fission. The relationship between two steps of Dex-protecting mitochondrial fission needs further investigation.

Although our experiments demonstrated that Dex protected the vascular endothelial barrier function by inhibiting mitochondrial division, there are some limitations that require further investigation. First, the present study verified the protective role of Dex in septic rats, and LPS stimulated VECs, but these models cannot fully simulate the complex pathophysiological environment in the human body. It is unknown whether Dex has a consistent protective function in large animals and humans. Second, We didn’t investigate the mechanism of ERK1/2 inhibition regulated by Dex, which some studies suggest is independent of α_2_-adrenoceptor but related to TLR4/MyD88/MAPK/NF-κB signaling pathway (Zhang et al., 2015). The detailed mechanisms need further exploration in the future. Moreover, we demonstrated the protective effect of Dex on VECs. However, in sepsis, the vascular smooth muscle cells (VSMCs) are less responsive to vasoactive drugs. Whether Dex can improve VSMCs responsiveness to vasoactive drugs and the mechanism must be further researched.

**FIGURE 7:**
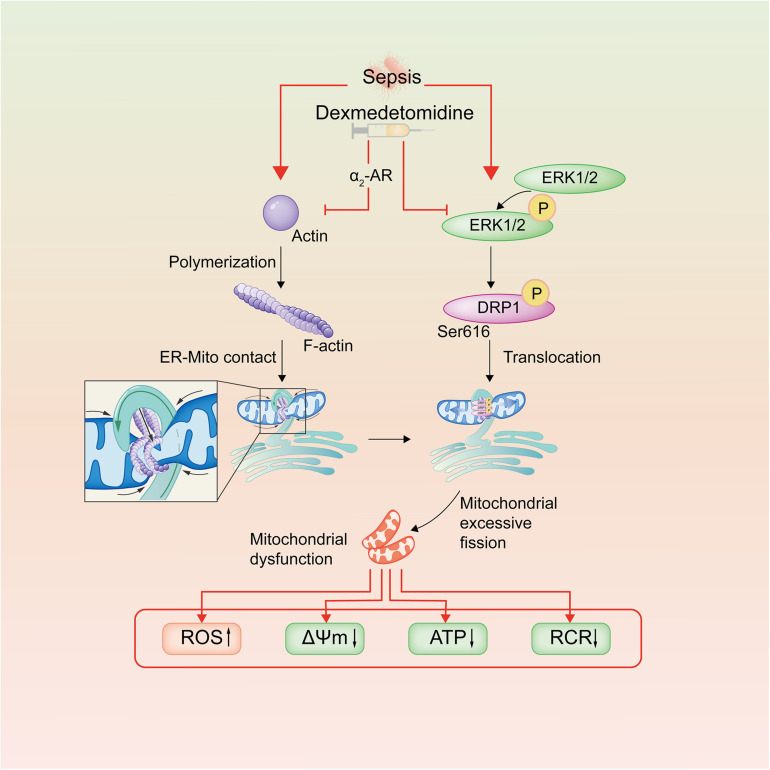
Schematic diagram of dexmedetomidine regulating mitochondrial fission pathways after sepsis. Dexmedetomidine could inhibite actin polymerization of VECs, leading to the decrease of ER-MITO contact sites. Meanwhile, it also down-regulated the phosphorylation of ERK1/2 and Drp1 ser616, leading to the decrease of the mitochondrial translocation of Drp1 after sepsis in VECs.

In conclusion, the present study demonstrated that Dex has a significant protective effect on the vascular endothelial barrier function of septic rats. The mechanism is related to inhibition of mitochondrial fission and protection of mitochondrial function through regulating the phosphorylation of Drp1 and reducing ER-MITO contacts of VECs via reducing actin polymerization (Figure 7). This research provides a potential therapeutic target for the treatment of sepsis.

## Data Availability Statement

The raw data supporting the conclusions of this article will be made available by the authors, without undue reservation.

## Ethics Statement

The animal study was reviewed and approved by the Laboratory Animal Welfare and Ethics Committee of the Army Medical University.

## Author Contributions

TL, YH, LL, and HS conceived and designed the study and analyzed the data. HS and HD drafted the manuscript. TL and YZ revised the manuscript. TL acquired the financial support. All authors performed the experimental procedures.

## Conflict of Interest

The authors declare that the research was conducted in the absence of any commercial or financial relationships that could be construed as a potential conflict of interest.

## Footnotes

1 https://fiji.sc/

2 http://igps.biocuckoo.org/down.php

## Supplementary Material

Supplementary Figure 1 The effects of dexmedetomidine on the mitochondrial fusion of vascular endothelial cells after sepsis. **(A–D)** Western blot analysis of OPA1, Mfn1 and Mfn2 in VECs after sepsis, *n* = 3. Normal, normal group; LPS, lps group; Dex, dexmedetomidine group.

## References

[B1] Allen J. M. Field C. Shoulders B. R. Voils S. A. (2019). Recent updates in the pharmacological management of sepsis and septic shock: a systematic review focused on fluid resuscitation, vasopressors, and corticosteroids. *Ann. Pharmacother.* 53 385–395. 10.1177/1060028018812940 30404539

[B2] Armstrong S. M. Mubareka S. Lee W. L. (2013). The lung microvascular endothelium as a therapeutic target in severe influenza. *Antiviral Res.* 99 113–118. 10.1016/j.antiviral.2013.05.003 23685311

[B3] Brooks C. Wei Q. Cho S. G. Dong Z. (2009). Regulation of mitochondrial dynamics in acute kidney injury in cell culture and rodent models. *J. Clin. Invest*. 119 1275–1285. 10.1172/JCI37829 19349686 PMC2673870

[B4] Chakrabarti R. Ji W. K. Stan R. V. de juan Sanz J. Ryan T. A. Higgs H. N. (2018). INF2-mediated actin polymerization at the ER stimulates mitochondrial calcium uptake, inner membrane constriction, and division. *J. Cell Biol.* 217 251–268. 10.1083/jcb.201709111 29142021 PMC5748994

[B5] Duan C. Kuang L. Xiang X. Zhang J. Zhu Y. Wu Y. (2020). Activated Drp1-mediated mitochondrial ROS influence the gut microbiome and intestinal barrier after hemorrhagic shock. *Aging* 12 1397–1416. 10.18632/aging.102690 31954373 PMC7053642

[B6] Duan C. Zhang J. Wu H. L. Li T. Liu L. M. (2017). Regulatory mechanisms, prophylaxis and treatment of vascular leakage following severe trauma and shock. *Mol. Med. Res.* 4:11. 10.1186/s40779-017-0117-6 28361006 PMC5370457

[B7] Dudek S. M. Garcia J. G. (2001). Cytoskeletal regulation of pulmonary vascular permeability. *J. Appl. Physiol.* 91 1487–1500. 10.1152/jappl.2001.91.4.1487 11568129

[B8] Eismann T. Huber N. Shin T. Kuboki S. Galloway E. Wyder M. (2009). Peroxiredoxin-6 protects against mitochondrial dysfunction and liver injury during ischemia-reperfusion in mice. *Am. J. Physiol. Gastrointest. Liver Physiol*. 296 266–274. 10.1152/ajpgi.90583.2008 19033532 PMC2643922

[B9] Friedman J. R. Lackner L. L. West M. Dibenedetto J. R. Nunnari J. Voeltz G. K. (2011). ER tubules mark sites of mitochondrial division. *Science* 334 358–362. 10.1126/science.1207385 21885730 PMC3366560

[B10] Gao Z. J. Li Y. Y. Wang F. Huang T. Fan K. Q. Zhang Y. (2017). Mitochondrial dynamics controls anti-tumour innate immunity by regulating CHIP-IRF1 axis stability. *Nat. Commun.* 8:1805. 10.1038/s41467-017-01919-0 29180626 PMC5703766

[B11] Hall A. M. Rhodes G. J. Sandoval R. M. Corridon P. R. Molitoris B. A. (2013). In vivo multiphoton imaging of mitochondrial structure and function during acute kidney injury. *Kidney Int*. 83 72–83. 10.1038/ki.2012.328 22992467 PMC4136483

[B12] Hatch A. L. Gurel P. S. Higgs H. N. (2014). Novel roles for actin in mitochondrial fission. *J. Cell Sci*. 127 4549–4560. 10.1242/jcs.153791 25217628 PMC4215709

[B13] Jawad I. Luksic I. Rafnsson S. B. (2012). Assessing available information on the burden of sepsis: global estimates of incidence, prevalence and mortality. *J. Glob. Health* 2:010404. 10.7189/jogh.02.010404 23198133 PMC3484761

[B14] Jiang H. K. Wang Y. H. Sun L. He X. Zhao M. Feng Z. H. (2014). Aerobic interval training attenuates mitochondrial dysfunction in rats post-myocardial infarction: roles of mitochondrial network dynamics. *Int. J. Mol. Sci*. 15 5304–5322. 10.3390/ijms15045304 24675698 PMC4013565

[B15] Jiang L. Li L. Shen J. Qi Z. Guo L. (2014). Effect of dexmedetomidine on lung ischemia-reperfusion injury. *Mol. Med. Rep.* 9 419–426. 10.3892/mmr.2013.1867 24345905 PMC3896524

[B16] Koh I. H. Menchaca-Diaz J. L. Koh T. H. Souza R. L. Shu C. M. Rogerio V. E. (2010). Microcirculatory evaluation in sepsis: a difficult task. *Shock* 34 27–33. 10.1097/SHK.0b013e3181e7e80c 20523273

[B17] Liu Y. Kalogeris T. Wang M. Zuidema M. Y. Wang Q. Dai H. (2012). Hydrogen sulfide preconditioning or neutrophil depletion attenuates ischemia-reperfusion-induced mitochondrial dysfunction in rat small intestine. *Am. J. Physiol. Gastrointest. Liver Physiol*. 302 44–54. 10.1152/ajpgi.00413.2010 21921289 PMC3345957

[B18] Miranda M. L. Balarini M. M. Bouskela E. (2015). Dexmedetomidine attenuates the microcirculatory derangements evoked by experimental Sepsis. *Anesthesiology* 122 619–630. 10.1097/ALN.0000000000000491 25313879

[B19] Nelson L. E. Lu J. Guo T. Z. Saper C. B. Franks N. P. Maze M. (2003). The alpha2-adrenoceptor agonist dexmedetomidine converges on an endogenous sleep-promoting pathway to exert its sedative effects. *Anesthesiology* 98 428–436. 10.1097/00000542-200302000-00024 12552203

[B20] Page A. V. Liles W. C. (2013). Biomarkers of endothelial activation/dysfunction in infectious diseases. *Virulence* 4 507–516. 10.4161/viru.24530 23669075 PMC5359744

[B21] Peake S. L. Delaney A. Bailey M. Bellomo R. Cameron P. A. Copper D. J. (2014). Goal-directed resuscitation for patients with early septic shock. *N. Engl. J. Med*. 371 1496–1506. 10.1056/NEJMoa1404380 25272316

[B22] Prieto J. Leon M. Ponsoda X. Sendra R. Bort R. Ferrer-Lorente R. (2016). Early ERK1/2 activation promotes DRP l-dependent mitochondrial fission necessary for cell reprogmmming. *Nat. Commun*. 7:11124. 10.1038/ncomms11124 27030341 PMC4821885

[B23] Qiu Z. Lu P. Wang K. Zhao X. Li Q. Wen J. (2020). Dexmedetomidine inhibits neuroinflammation by altering microglial M1/M2 polarization through MAPK/ERK pathway. *Neurochem. Res.* 45 345–353. 10.1007/s11064-019-02922-1 31823113

[B24] Rowland A. A. Vorletz G. K. (2012). Endoplasmic reticulum-mitochondria contacts: function of the junction. *Nat. Rev*. 13 601–615. 10.1038/nrm3440 22992592 PMC5111635

[B25] Roy S. Kim D. Sankaramoorthy A. (2019). Mitochondrial structural changes in the pathogenesis of diabetic retinopathy. *J. Clin. Med.* 8:1363. 10.3390/jcm8091363 31480638 PMC6780143

[B26] Schmidt K. Hernekamp J. F. Philipsenburg C. Zivkovic A. R. Brenner T. Hofer S. (2015). Time-dependent effect of clonidine on microvascular permeability during endotoxemia. *Microvasc. Res*. 101 111–117. 10.1016/j.mvr.2015.07.002 26177515

[B27] Sha J. Zhang H. Zhao Y. Feng X. Hu X. Wang C. (2019). Dexmedetomidine attenuates lipopolysaccharide-induced liver oxidative stress and cell apoptosis in rats by increasing GSK-3β/MKP-1/Nrf2 pathway activity via the α_2_ adrenergic receptor. *Toxicol. Appl. Pharmacol*. 364 144–152. 10.1016/j.taap.2018.12.017 30597158

[B28] Shinh H. W. Shinotsuka C. Torii S. Murakami K. Nakayama K. (1997). Identification and subcellular localization of a novel mammalian dynamin-related protein homologous to yeast Vps1p and Dnm1p. *J. Biochem*. 122 525–530. 10.1093/oxfordjournals.jbchem.a021784 9348079

[B29] Sun Y. B. Zhao H. Mu D. L. Zhang W. Cui J. Wu l. (2019). Dexmedetomidine inhibits astrocyte pyroptosis and subsequently protects the brain in in vitro and in vivo models of sepsis. *Cell Death Dis*. 10:167. 10.1038/s41419-019-1416-5 30778043 PMC6379430

[B30] Valente A. J. Maddalena L. A. Robb E. l. Moradi F. Stuart J. A. (2017). A simple ImageJ macro tool for analyzing mitochondrial network morphology in mammalian cell culture. *Acta Histochem*. 119 315–326. 10.1016/j.acthis.2017.03.001 28314612

[B31] Vincent J. L. Opal S. M. Marshall J. C. Tracey K. J. (2013). Sepsis definitions: time for change. *Lancet* 381 774–775. 10.1016/S0140-6736(12)61815-7 23472921 PMC4535310

[B32] Wales P. Schuberth C. E. Aufschnaiter R. Fels J. Garcia-Aguilar L. Janning A. (2016). Calcium-mediated actin reset (CaAR) mediates acute cell adaptations. *Elife* 5:19850. 10.7554/eLife.19850 27919320 PMC5140269

[B33] Wang C. W. Shui K. Ma S. S. Lin S. F. Zhang Y. Wen B. (2020). Integrated omics in Drosophila uncover a circadian kinome. *Nat.Commun.* 11:2710. 10.1038/s41467-020-16514-z 32483184 PMC7264355

[B34] Wang Y. Mattson M. P. Furukawa K. (2002). Endoplasmic reticulum calcium release is modulated by actin polymerization. *J. Neurochem.* 82 945–952. 10.1046/j.1471-4159.2002.01059.x 12358800

[B35] Wu W. Lin C. Wu K. Jiang L. Wang X. Li W. (2016). FUNDC1 regulates mitochondrial dynamics at the ER-mitochondrial contact site under hypoxic conditions. *EMBO J.* 35 1368–1384. 10.15252/embj.201593102 27145933 PMC4864280

[B36] Yuan F. Fu H. Sun K. Wu S. Dong T. (2017). Effect of dexmedetomidine on cerebral ischemia-reperfusion rats by activating mitochondrial ATP-sensitive potassium channel. *Metab. Brain Dis*. 32 539–546. 10.1007/s11011-016-9945-4 28035625

[B37] Zhang J. Wang J. Luan T. Zuo Y. Chen J. Zhang H. (2019). Deubiquitinase USP9X regulates the invasion of prostate cancer cells by regulating the ERK pathway and mitochondrial dynamics. *Oncol. Rep.* 41 3292–3304. 10.3892/or.2019.7131 31002345 PMC6489063

[B38] Zhang J. Wang Z. Wang Y. Zhou G. Li H. (2015). The effect of dexmedetomidine on inflammatory response of septic rats. *BMC Anesthesiol.* 15:68. 10.1186/s12871-015-0042-8 25929655 PMC4422264

[B39] Zhao H. Zhu Y. Zhang J. Wu Y. Xiang X. Zhang Z. (2020). The beneficial effect of HES on vascular permeability and its relationship with endothelial glycocalyx and intercellular junction after hemorrhagic shock. *Front. Pharmacol.* 11:597. 10.3389/fphar.2020.00597 32457611 PMC7227604

[B40] Zhao J. Zhou C. L. Xia Z. Y. Wang L. (2013). Effects of dexmedetomidine on L-Type calcium current in rat ventricular myocytes. *Acta Cardiol. Sin.* 29 175–180. 10.1016/j.pcad.2013.01.003 27122702 PMC4804780

[B41] Zhao L. Zhuang J. Wang Y. Zhou D. Zhao D. Zhu S. (2019). Propofol ameliorates H9C2 cells apoptosis induced by oxygen glucose deprivation and reperfusion injury via inhibiting high levels of mitochondrial fusion and fission. *Front. Pharmacol.* 10:61. 10.3389/fphar.2019.00061 30809145 PMC6379462

[B42] Zheng D. Zhang J. Zhang Z. Kuang L. Zhu Y. Wu Y. (2020). Endothelial microvesicles induce pulmonary vascular leakage and lung injury during sepsis. *Front. Cell Dev. Biol*. 8:643. 10.3389/fcell.2020.00643 32766250 PMC7379030

[B43] Zhu Y. Wu H. Wu Y. Zhang J. Peng X. Zang J. (2016). Beneficial effect of intermedin 1-53 in septic shock rats: contributions of Rho kinase and Bkca pathway-mediated improvement in cardiac function. *Shock* 46 557–565. 10.1097/SHK.0000000000000639 27355401

[B44] Zi S. F. Li J. H. Liu L. Deng C. Ao X. Chen D. D. (2019). Dexmedetomidine-mediated protection against septic liver injury depends on TLR4/MyD88/NF-κB signaling downregulation partly via cholinergic anti-inflammatory mechanisms. *Int. Immunopharmacol*. 76:105898. 10.1016/j.intimp.2019.105898 31520992

