# Supplementary figures and images for: Protective Effects of Dexmedetomidine on the Vascular Endothelial Barrier Function by Inhibiting Mitochondrial Fission via ER/Mitochondria Contact
Source: Front Cell Dev Biol. 2021 Mar 11;9:636327. doi: 10.3389/fcell.2021.636327 (PMC7991806; doi:10.3389/fcell.2021.636327)

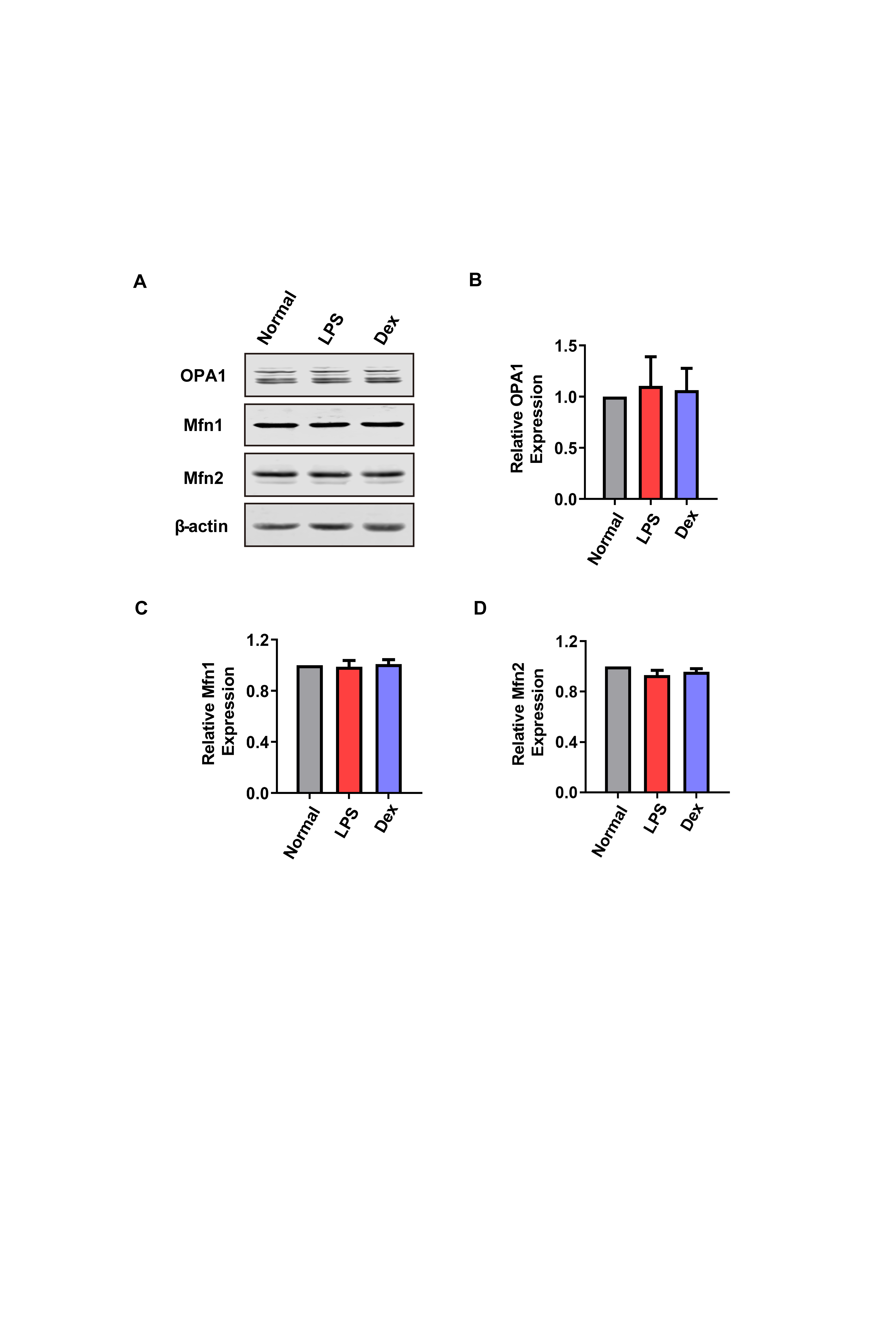

Supplement: Supplementary Figure 1 — The effects of dexmedetomidine on the mitochondrial fusion of vascular endothelial cells after sepsis. (A–D) Western blot analysis of OPA1, Mfn1 and Mfn2 in VECs after sepsis, n = 3. Normal, normal group; LPS, lps group; Dex, dexmedetomidine group. [file Image_1.TIF]
